# Environmental and evolutionary drivers of diversity patterns in the tea family (Theaceae s.s.) across China

**DOI:** 10.1002/ece3.4619

**Published:** 2018-11-08

**Authors:** Mide Rao, Manuel J. Steinbauer, Xiaoguo Xiang, Minggang Zhang, Xiangcheng Mi, Jintun Zhang, Keping Ma, Jens‐Christian Svenning

**Affiliations:** ^1^ Key Laboratory of Biodiversity Sciences and Ecological Engineering, Ministry of Education, College of Life Sciences Beijing Normal University Beijing China; ^2^ State Key Laboratory of Vegetation and Environmental Change, Institute of Botany Chinese Academy of Sciences Beijing China; ^3^ Section for Ecoinformatics and Biodiversity, Department of Bioscience Aarhus University Aarhus C Denmark; ^4^ State Key Laboratory of Systematic and Evolutionary Botany, Institute of Botany Chinese Academy of Sciences Beijing China; ^5^ Institute of Loess Plateau Shanxi University Taiyuan China; ^6^ Center for Biodiversity Dynamics in a Changing World (BIOCHANGE) Aarhus University Aarhus C Denmark

**Keywords:** China, evolution, phylogeny, soil pH, species richness, Theaceae

## Abstract

Subtropical forest is recognized as an important global vegetation type with high levels of plant species richness. However, the mechanisms underlying its diversity remain poorly understood. Here, we assessed the roles of environmental drivers and evolutionary dynamics (time‐for‐speciation and diversification rate) in shaping species richness patterns across China for a major subtropical plant group, the tea family (Theaceae s.s.) (145 species), at several taxonomic scales. To this end, we assessed the relationships between species richness, key environmental variables (minimum temperature of the coldest month, mean annual precipitation, soil pH), and phylogenetic assemblage structure (net related index) by using non‐spatial and spatial linear models. We found that species richness is significantly related to environmental variables, especially soil pH, which is negatively related to species richness both across the whole family and within the major tribe Theeae (116 species). Family‐level species richness is unrelated to phylogenetic structure, whereas species richness in tribe Theeae was related to phylogenetic structure with U‐shaped relationship, a more complex relation than predicted by the time‐for‐speciation or diversification rate hypotheses. Overall, these results suggest that both environmental and evolutionary factors play important roles in shaping species richness patterns within this subtropical plant family across China, with the latter mainly important at fine taxonomic scales. Most surprisingly, our findings show that soils can play a key role in shaping macro‐scale diversity patterns, contrary to often‐stated assumptions.

## 1 INTRODUCTION

Subtropical forests are important terrestrial ecosystems that contribute importantly to both biodiversity and ecosystem functioning (Fang & Yoda, 1991; Wu & Wu, 1998). This is especially true in China, which contains the largest subtropical forest area in the world (Song, Chen, & Wang, 2005; Zhu, 2013; Zhu et al., 2008). Although many studies have explored drivers of high diversity in the tropics (Mittelbach et al., 2007; Wiens & Donoghue, 2004), what drives diversity patterns within the large subtropical region in China remains poorly understood.

Climate is typically expected to be the primary driver of species diversity patterns at large geographic scales (Field et al., 2009; Hawkins et al., 2003). Increasing intensity of frost has been found to determine the decline in woody species richness with latitude in China, with annual precipitation also explaining much of the variation in woody species richness (Wang, Fang, Tang, & Lin, 2011). Subtropical China is characterized by the monsoon climate with mean annual temperature ranging from 15 to 20°C, mean annual precipitation (MAP) ranging from 900 to 2,000 mm, and a frost period ranging from 64 to 100 days (Wang, Kent, & Fang, 2007). We thus expect that species diversity patterns within this region are shaped by both rainfall and temperature. However, environmental variables related to edaphic properties, such as soil pH, are suggested as important predictors for species richness at local scales (Palpurina et al., 2017; Pausas & Austin, 2001; Zellweger et al., 2016). Subtropical China is mainly covered with red soils, which are highly weathered, nutrient‐deficient, and acidic with high accumulation of aluminum (Al) and iron (Fe) (Wilson, He, & Yang, 2004). Soil pH strongly affects the exchangeable base cations (calcium [Ca], magnesium [Mg], potassium [K], sodium [Na]), mobilizing exchangeable Al and affecting the availability of nutrients such as K and phosphorus (P), which are crucial for tree growth (Lieb, Darrouzet‐Nardi, & Bowman, 2011). Soil pH displays a strong trend, increasing from the southeast to the northwest across China (Xiong & Li, 1987), which suggests that it could play a role in large‐scale diversity patterns. However, the role of soil pH in shaping species richness at large spatial scales has rarely been studied neither generally nor for China specifically (but see Azevedo, Zelm, Hendriks, Bobbink, & Huijbregts, 2013).

Patterns of species richness at large spatial scales should ultimately be associated with evolutionary and biogeographic processes that directly influence the number of species, namely, speciation, extinction, and dispersal (Ricklefs, 2004; Wiens, Parra‐Olea, Garcia‐Paris, & Wake, 2007). Two general explanations have been proposed to explain geographical patterns in species richness (Wiens, 2011). First, species‐rich areas may have had more time for speciation to accumulate species due to longer occupancy in certain environmental conditions (Qian, Wiens, Zhang, & Zhang, 2015; Stephens & Wiens, 2003). Second, more species‐rich areas may have experienced higher diversification (i.e., speciation minus extinction) rates in certain environmental conditions (Qian et al., 2015; Svenning, Borchsenius, Bjorholm, & Balslev, 2008). Environmental factors could play different roles underlying these two explanations: Under the first hypothesis, known as time‐for‐speciation, environmental factors are associated with limitations to the colonization of new habitats due to niche conservatism (Qian, Zhang, Zhang, & Wang, 2013; Wiens & Donoghue, 2004; Wiens et al., 2010). Under the second hypothesis, called diversification rate, environmental factors are responsible for species richness patterns through their influence on the diversification rate (Qian et al., 2015; Wiens, 2011). Evidence for time‐for‐speciation has been found for various taxonomic groups (Kerkhoff, Moriarty, & Weiser, 2014; Li et al., 2009; Wiens, Graham, Moen, Smith, & Reeder, 2006). The diversification rate hypothesis also has been supported by several studies (Cardillo, Orme, & Owens, 2005; Pyron & Wiens, 2013; Svenning et al., 2008). Consequently, these mechanisms are not mutually exclusive and their relative importance may vary by clade and region. Relatively few studies have tested these two hypotheses simultaneously (Marin & Hedges, 2016; Qian et al., 2015; Svenning et al., 2008), particularly in subtropical regions.

The tea family (Theaceae s.s.) is a dominant woody constituent of subtropical forests in eastern Asia (Wu, 1995). Theaceae s.s. includes three major lineages: tribes Theeae, Gordonieae, and Stewartieae, with Theeae being the most species‐rich. Theaceae species are concentrated in subtropical forests and tropical mountain areas, with most genera being evergreen broad‐leaved shrubs and trees (Luna & Ochoterena, 2004). Their geographic distribution and characteristic traits reflect their limited tolerance of frost (Sakai & Weiser, 1973). Furthermore, Theaceae species usually require ample amounts of water and prefer acidic soils (Ming, 2000). There is a rich fossil record for Theaceae, beginning from the late Cretaceous through the Paleo‐ and the Neogene (Prince, 2009), which shows that Theaceae was a conspicuous component of the vegetation across North America, Europe, and Asia prior to the late Neogene cooling and the Quaternary glaciations (Grote & Dilcher, 1989). Today, Theaceae s.s. diversity is concentrated in southeast Asia and in southern China and is absent from Europe (Grote & Dilcher, 1989).

In this study, we combine species richness, environmental and phylogenetic data for Theaceae s.s. to address the following questions: (a) How are the patterns of species richness in Theaceae s.s. distributed along environmental gradients across China? (b) What causes these patterns? Is the higher species richness in some environments caused by greater time for building up species or/and faster net rates of diversification in certain environments? (c) Do the causes of the species richness patterns differ at the family and tribe levels?

## 2 MATERIALS AND METHODS

### 2.1 Species distribution and environmental data

We compiled presence/absence data for Theaceae s.s. (hereafter Theaceae for simplicity) (following *Flora of China,* Ming & Bartholomew, 2007) in 100 km × 100 km cells across China using the Chinese Vascular Plant Database, in which plant occurrence locations were recorded at the county level. This database is based on: (a) a specimen's locality from the National Specimen Information Infrastructure ( www.nsii.org.cn); (b) published and provincial floras, including *Flora Republicae Popularis Sinicae* (FRPS: Flora of China, Chinese version); as well as (c) public checklists, and species surveying reports for all 145 Chinese tea family species (117 in Theeae, 13 in Gordonieae, 15 in Stewartieae). We removed border cells with an area <5,000 km^2^. Because the sample sizes for Gordonieae and Stewartieae were too small, analyses were only run on for the full dataset, comprising the whole family (Theaceae), and for the biggest tribe (Theeae). To compute the phylogenetic assemblage structure (see below), cells with fewer than three Theaceae or Theeae species were excluded, yielding a final subset of 233 cells and 145 species for the family‐wide dataset and of 230 cells and 116 species for the tribe‐level dataset.

Environmental data were sampled for all grid cells in each subset. Nineteen bioclimatic variables were obtained from WorldClim (Hijmans, Cameron, Parra, Jones, & Jarvis, 2005), but we mainly used minimum temperature of the coldest month (MINT) and MAP. We excluded other variables due to their weak predictive abilities or because they were highly correlated with MINT/MAP in our preliminary analysis. Soil pH was obtained from SoilGrids (Hengl et al., 2014). All data were obtained at a resolution of 30 arc seconds.

### 2.2 Phylogenetic analysis

We sampled a total of 133 species with relevant sequences from GenBank, including 123 ingroup and 10 outgroup species, which were selected from closely related families in Styracaceae and Symplocaceae (see Supporting Information Table S1). The taxonomy of species in China follows Ming and Bartholomew (2007), whereas the taxonomy of species outside of China was based on The Plant List ( https://www.theplantlist.org). Ten chloroplast DNA sequences (*atpB‐rbcL, atpI‐atpH, matK, matK‐trnK, psbA‐trnH, rbcL, rbcL‐accD, rpl16, rpl32‐trnL, and trnL‐trnF*) and the nuclear internal transcribed spacer (ITS) region were used for analyses (GenBank accession numbers are given in Supporting Information Table S1).

Sequences alignment was performed in Clustal X (Thompson, Gibson, Plewniak, Jeanmougin, & Higgins, 1997) and then adjusted manually in BioEdit (Hall, 1999). The cpDNA and ITS regions were analyzed separately. The homogeneity between the cpDNA and ITS was tested using the incongruence length difference test (Farris, Källersjö, Kluge, & Bult, 1994), which was implemented in PAUP v4.0b10 (Swofford, 2003). The result (*p* = 0.01) suggested the incongruence between ITS and cpDNA. According to Nishii et al. (2015), incongruences supported by bootstrap values higher than 75% and/or 0.95 posterior probabilities would be regarded as significant. A comparison of the topologies of the ITS with the cpDNA trees for the analyses revealed no strong conflict in relationships between the main clades. Thus, the combined cpDNA and ITS matrix was used to estimate the divergence times.

**Figure 1:**
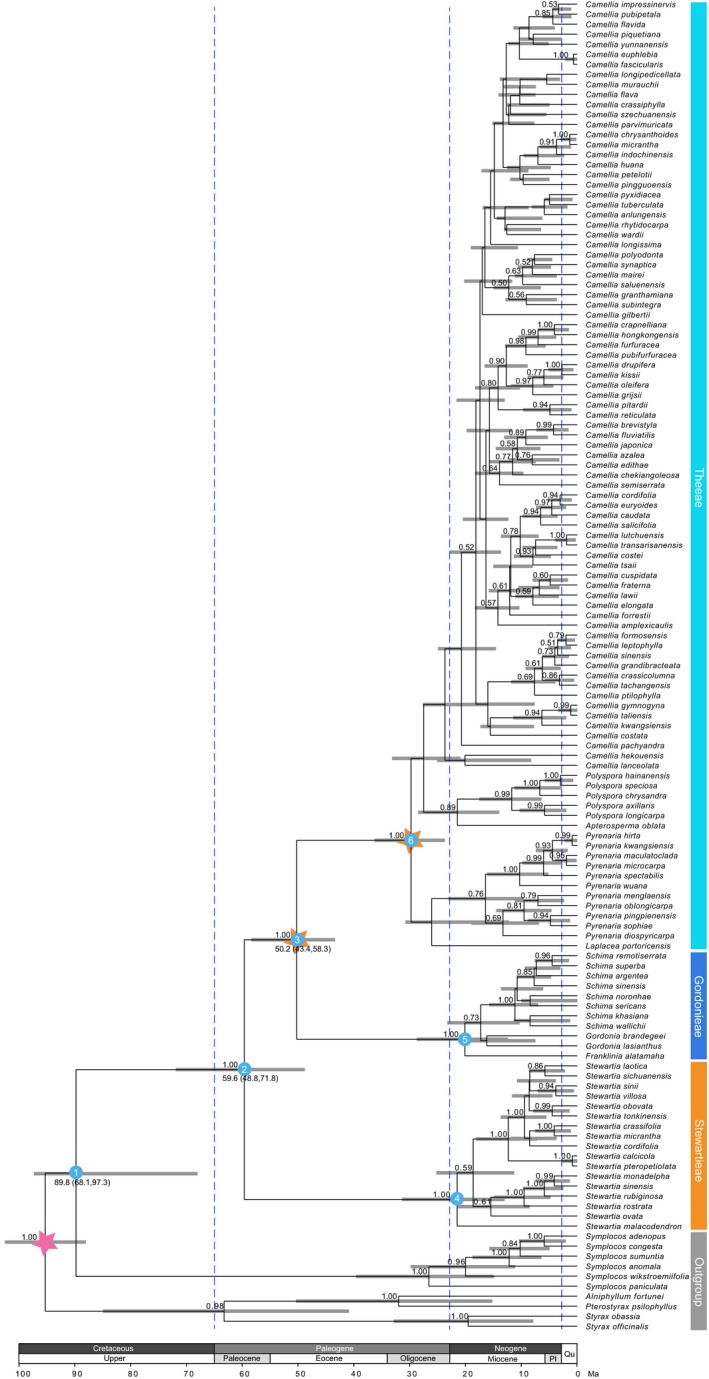
Timing of the diversification of Theaceae (133 species). Chronogram is derived from the maximum clade credibility tree by BEAST analyses. Gray bars indicate 95% highest posterior density (HPD) intervals of the age estimates. The red and orange stars represent the root and fossil calibration nodes separately (see Section 2). The blue circles with numbers indicate the nodes of interest (see Section 3). Numbers above the branch indicate Bayesian inference (BI) posterior probability (PP) values. Only nodes with PP support >0.5 are shown. Numbers below the branch indicate the estimated divergent median ages for the nodes, where values in parentheses represent 95% HPD. The ages of stratigraphic boundaries were obtained from the International Chronostratigraphic Chart (Cohen, Finney, Gibbard, & Fan, 2013) (Pl, Pliocene; Qu, Quaternary)

**Figure 2:**
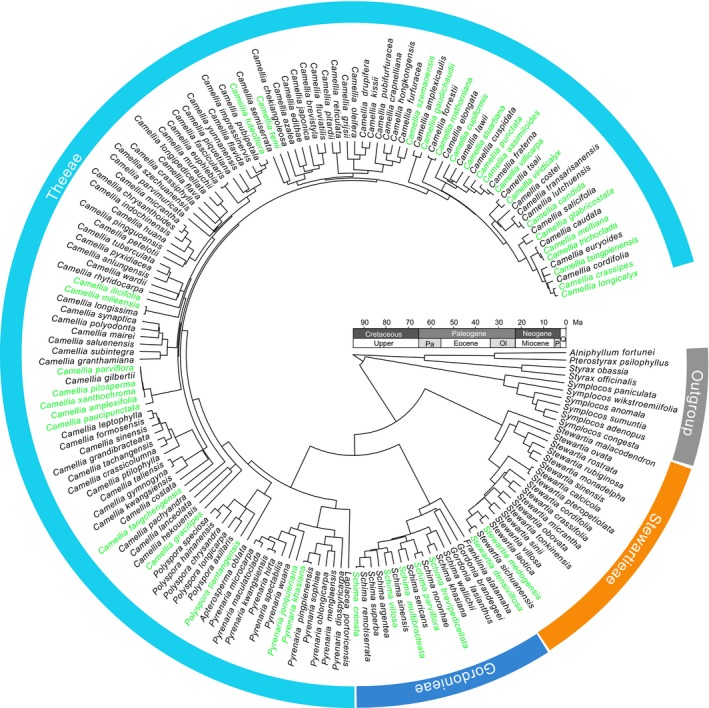
Example of a dated tree of Theaceae (170 species) from the set of 1,000 trees where species without genetic information have been added with random branch lengths in their responding sections, respectively. Black tip labels indicate sequenced species. Green tip labels indicate non‐sequenced species. The ages of stratigraphic boundaries were obtained from the International Chronostratigraphic Chart (Cohen et al., 2013) (Pa, Paleocene; Ol, Oligocene; P, Pliocene; Qu, Quaternary)

Divergence times were estimated using a Bayesian uncorrelated relaxed‐clock model in BEAST 2.3.0 (Bouckaert et al., 2014). For BEAST analysis, cpDNA and ITS were assigned GTR+I+Γ, respectively, as determined by the Akaike information criterion (AIC) in Modeltest 3.7 (Posada & Crandall, 1998). Three calibration points were used: the root was set to 96 million years ago (Ma) with a normal prior distribution for the whole tree (88–103 Ma) (Wikstrom, Kainulainen, Razafimandimbison, Smedmark, & Bremer, 2015) (Figure 1); the stem node and crown node of Theeae were constrained to a minimum age of 40 Ma (Figure 1) and 20 Ma (Figure 1), respectively, using a lognormal prior distribution with a standard deviation of 1.0, and following the example of Zhang, Kan, Zhao, Li, and Wang (2014). The Yule process was chosen as the speciation process. Markov chain Monte Carlo searches were run for 100,000,000 generations and sampled every 1,000 generations. Convergence and effective sample sizes of all parameters were assessed in Tracer 1.6.0 (Rambaut, Suchard, Xie, & Drummond, 2014). The maximum clade credibility tree was computed using treeAnnotator 2.3.0 (Bouckaert et al., 2014) and is shown in Figure 1 (133 species). Thirty‐seven species in China that did not have molecular data were added manually to the tree as follows: (a) We randomly selected 1,000 trees from the BEAST tree set after the burn‐in; (b) based on known morphological classification information from the Flora of China (Ming & Bartholomew, 2007), we added the 37 species to each tree in their corresponding sections with random branch lengths using the R package “phytools” (Revell, 2012). Finally, we had compiled a set of 1,000 random‐addition trees with 170 species (for example tree, see Figure 2).

We calculated the mean phylogenetic net relatedness index (NRI; Webb, 2000) based on the set of 1,000 random‐addition trees and quantified the degree of phylogenetic relatedness among species for each 100 km × 100 km cell. NRI was calculated using the mean pairwise distance (MPD), which measures the mean phylogenetic relatedness between all pairs of species occurring in an assemblage: NRI = −1 × (MPD_observed_ – MPD_randomized_)/sd(MPD_randomized_), where MPD_observed_ is calculated from species occurring in the given cell, and MPD_randomized_ is the expected MPD distribution from the 1,000 null models. Positive values of NRI indicate that the species present in an assemblage are more closely related to each other than expected by chance (phylogenetic clustering), while negative values of NRI indicate that the species are less related to each other than expected by chance (phylogenetic overdispersion). Because the values are centered at zero and standardized by the standard deviation, values >1.96 indicate statistically significant phylogenetic clustering, while values <−1.96 indicate statistically significant phylogenetic overdispersion (Vamosi, Heard, Vamosi, & Webb, 2009). NRI was calculated in R 3.3.2 (R Core Team, 2016) with the “picante” package (Kembel et al., 2010).

### 2.3 Statistical analyses

We first used Pearson correlations to assess pairwise relationships among the different variables. To account for spatial autocorrelation, Dutilleul's (1993) modified *t* test was used to calculate statistical significance with package “SpatialPack 0.2‐3” (Osorio & Vallejos, 2014) in R.

Spatial linear models (SLM) were applied to estimate the richness patterns of tea family species along environmental gradients (MINT, MAP, pH) across China. To account for nonlinear relationships between the variables, the quadratic terms of the predictor variables were included in the regressions. To avoid multicollinearity problems, we divided environmental variables (i.e., MAP and soil pH, Tables 1 and 2) with high pairwise correlation (|*r*|>0.7) (Dormann et al., 2013) into different datasets for the modeling. Thus, we defined two groups of environmental variables that were only used separately: MINT+MAP and MINT+pH.

**Table 1:** Pearson correlations among variables for Theaceae

Variable	MINT	MAP	pH	NRI
MAP	**0.617 (0.046)**			
pH	**−**0.399 (0.141)	**−0.813 (0.035)**		
NRI	0.102 (0.263)	**−**0.034 (0.664)	0.145 (0.071)	
SR	**0.427 (0.031)**	0.424 (0.059)	**−0.512 (0.007)**	−0.067 (0.479)

Note

*P* values were calculated after accounting for spatial autocorrelation in parentheses. Significant values (*p* < 0.05) are marked in bold.

**Table 2:** Pearson correlations among variables for Theeae

Variable	MINT	MAP	pH	NRI
MAP	**0.587 (0.021)**			
pH	**−**0.364 (0.102)	**−0.804 (0.000)**		
NRI	**−**0.145 (0.221)	**−**0.119 (0.352)	0.199 (0.081)	
SR	**0.444 (0.019)**	0.366 (0.106)	**−0.397 (0.038)**	0.100 (0.316)

Note

*P* values were calculated after accounting for spatial autocorrelation in parentheses. Significant values (*p* < 0.05) are marked in bold.

We sought to identify the drivers of species richness along environmental gradients by comparing patterns of phylogenetic relatedness among species in different species assemblages and relating these patterns to environmental factors (Algar, Kerr, & Currie, 2009; Qian et al., 2015). For example, if time is important for patterns of species richness along environmental gradients, in species‐rich environments, species should be relatively distantly related to each other, indicating a long period of occupancy. Then for environments with rare and recent colonization, species should be relatively closely related (Qian et al., 2015, 2013 ). Therefore, a negative relationship between NRI and species richness, and a significant relationship between NRI and a certain environmental variable may support the time‐for‐speciation hypothesis. If higher richness in certain environments is much more likely caused by a higher rapid diversification rate, then species should be relatively more closely related to each other in high‐richness environments. Thus, a positive relationship between NRI and species richness, and a significant relationship between NRI and a certain environmental variable should be found (Qian et al., 2015). If both time and diversification rate are important in explaining species richness patterns, the different relationships between species richness and NRI may cancel each other out, resulting in weak or absent species richness–NRI relationships. Here, we used SLM to analyze the relationship between richness and NRI, as well as the relationships between NRI and different groups of environmental variables.

We also fitted non‐spatial ordinary least squares (OLS) regression models to complement the SLM results. Since significant spatial autocorrelation was found in the residuals of OLS models (Supporting Information Tables [Link], [Link], and S4), we here emphasize the SLM results. Still, we also report the OLS results, as spatial autocorrelation has been argued to not seriously affect OLS estimation of regression coefficients (Hawkins, Diniz‐Filho, Mauricio Bini, Marco, & Blackburn, 2007), and that controlling for spatial autocorrelation may shift the spatial scale of the analyses (Diniz‐Filho, Bini, & Hawkins, 2003). The OLS model residuals were found to approximate a normal distribution (Supporting Information Figure S1).

Both SLM and OLS models were run in R. For SLM, spatial simultaneous autoregressive error (SAR) models were built using the “spdep” package (Bivand et al., 2015). The Moran's *I* values were used to quantify the presence of spatial autocorrelation in SAR or OLS models (Kissling & Carl, 2008). The best SAR or OLS models were identified based on the Akaike information criterion corrected for small sample size (AIC_c_) (Burnham & Anderson, 2002). To evaluate the relative importance of variables in the SAR or OLS modeling, the importance of each predictor was determined by Akaike weights, computed with the “MuMIn” package (Barton, 2015). Species richness and MAP were log‐transformed to improve normality in our models, and all predictor variables were standardized to a mean of zero and standard deviation of one to allow for the direct comparison of regression coefficients.

## 3 RESULTS

Within Theaceae, all three tribes (Stewartieae, Gordonieae, and Theeae) were found to be monophyletic with strong support values (BI = 1.0; nodes 4, 5, and 6; Figure 1). Stewartieae was the first divergent clade (BI = 1.0; node 2, Figure 1), while Gordonieae was sister to Theeae with strong support values (BI = 1.0; node 3, Figure 1). The stem age of Theaceae was estimated at 89.8 Ma (95% highest posterior density (HPD): 68.1–97.3; node 1; Figure 1), and the crown age of the family was estimated to be 59.6 Ma (95% HPD: 48.8–71.8; node 2; Figure 1). Gordonieae and Theeae were estimated to have diverged at 50.2 Ma (95% HPD: 43.4–58.3; node 3; Figure 1).

**Figure 3:**
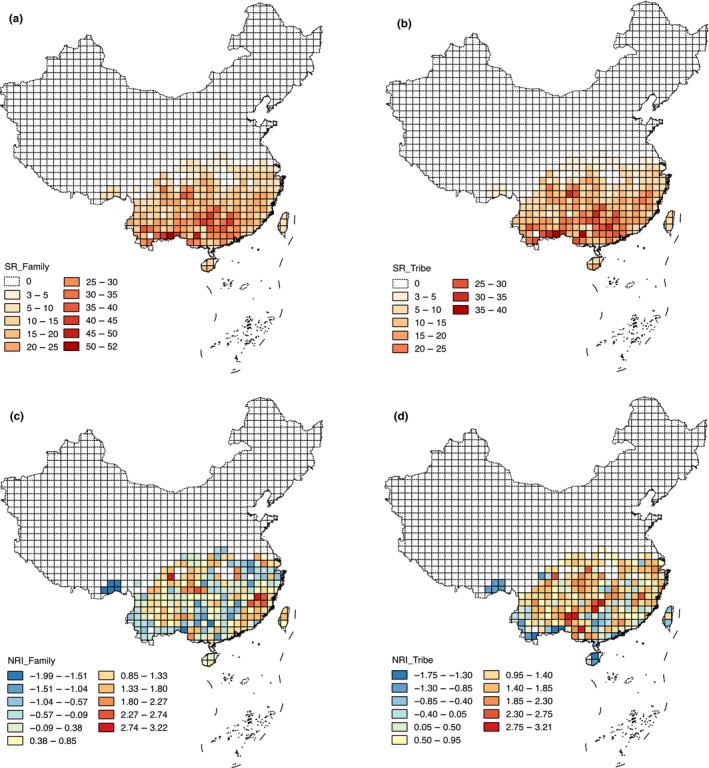
Geographical patterns of (a) species richness for Theaceae (SR_Family); (b) species richness for Theeae (SR_Tribe); (c) net relatedness index (NRI) for Theaceae (NRI_Family); (d) NRI for Theeae (NRI_Tribe)

The number of Theaceae species in the sampled quadrats generally decreased with latitude (Figure 3a), but also displayed more complex geographic variation (Figure 3b). Neither Theaceae nor Theeae showed clear geographic gradients in phylogenetic structure (NRI) (Figure 3c,d).

At family level, species richness was significantly correlated with both MINT (*r* = 0.427) and soil pH (*r *= −0.512) after accounting for spatial autocorrelation (*p* < 0.05). Similarly, species richness in the Theeae tribe was positively correlated with MINT (*r* = 0.444, *p* < 0.05) and negatively correlated with soil pH (*r *= −0.397, *p* < 0.001). Precipitation (MAP) was not correlated with species richness at either taxonomic level (Tables 1 and 2). Species richness and NRI were not related at either family level or tribe level (Tables 1 and 2). NRI was also not correlated with environmental variables at either level (Tables 1 and 2).

Among the SAR models for species richness, models including soil pH were much stronger than models including precipitation. Soil pH and species richness consistently had a strong negative relationship at both family level and tribe level (Table 3). MINT was negatively associated with species richness at family level, but the relationship was weak, and there was no effect of MINT on species richness at tribe level (Table 3).

**Table 3:** Multimodel inference from spatial simultaneous autoregressive error (SAR) models of species richness against environmental predictors for Theaceae and Theeae

Model parameters	Theaceae				Theeae			
	Coefficients	Akaike weight	Pseudo‐*r* ^2^	Moran's *I*	Coefficients	Akaike weight	Pseudo‐*r* ^2^	Moran's *I*
Group 1								
MINT	−0.084*	0.680	0.515	−0.023 *ns*	–		0.478	−0.024* ns*
MINT^2^	–				–			
MAP	0.167***	1.000			0.116***	1.000		
MAP^2^	−0.029*	0.770			–			
Group 2								
MINT	–		0.558	−0.024* ns*	–		0.514	−0.026* ns*
MINT^2^	–				–			
pH	−0.169***	1.000			−0.132***	1.000		
pH^2^	–				–			

Note

Model sets involved all possible combinations of explanatory variables, for two groups of variables: Group 1: minimum temperature of the coldest month (MINT), mean annual precipitation (MAP). Group 2: MINT, soil pH (pH). Coefficients for the model with the lowest AICc for a given variable group are shown. The Akaike weight for each variable is based on the full model set per group. The superscript 2 indicates the quadratic form of the variables. Pseudo‐*r* ^2^, explained the variance of the SAR model. Moran's *I*, measure of residual spatial autocorrelation.

Significance levels:

a *p* < 0.001;

*p* < 0.01;

b *p* < 0.05. *ns*, not significant.

According to the SAR models, species richness was independent of NRI at family level, but showed a U‐shaped relationship at tribe level (Table 4), suggesting an evolutionary link at this lower scale.

**Table 4:** Multimodel inference from spatial simultaneous autoregressive error (SAR) models of species richness against phylogenetic predictors for Theaceae and Theeae

Model parameters	Theaceae				Theeae			
	Coefficients	Akaike weight	Pseudo‐*r* ^2^	Moran's *I*	Coefficients	Akaike weight	Pseudo‐*r* ^2^	Moran's *I*
NRI	−0.017* ns*	0.400	0.441	−0.020 *ns*	–		0.491	−0.014* ns*
NRI^2^	–				0.037***	1.000		

Note

Coefficients for the model with the lowest AIC_c_ are shown. The Akaike weight for each variable is based on the full model set. The superscript 2 indicates the quadratic form of the variable. Pseudo‐*r* ^2^, explained the variance of the SAR model. Moran's *I*, measure of residual spatial autocorrelation.

Significance levels:

a *p* < 0.001;

*p* < 0.01;

*p* < 0.05. *ns*, not significant.

Among the SAR models for NRI, the MINT+pH model was a better fit than the MINT+MAP model at family level (Table 5). Soil pH was much more important than temperature for phylogenetic structure, as shown by the strong positive NRI‐pH relationship at family level. However, only MINT affected NRI at tribe level (Table 5), where MINT was unimodally related to NRI.

**Table 5:** Multimodel inference from spatial simultaneous autoregressive error (SAR) models of phylogenetic structure (NRI) against environmental predictors for Theaceae and Theeae

Model parameters	Theaceae				Theeae			
	Coefficients	Akaike weight	Pseudo‐*r* ^2^	Moran's *I*	Coefficients	Akaike weight	Pseudo‐*r* ^2^	Moran's *I*
Group 1								
MINT	0.264*	0.550	0.210	0.003* ns*	−0.230*	0.630	0.216	−0.004* ns*
MINT^2^	–				−0.153**	0.900		
MAP	−0.186* ns*	0.440			–			
MAP^2^	–				–			
Group 2								
MINT	0.212* ns*	0.590	0.226	0.002* ns*	−0.230*	0.660	0.216	−0.004* ns*
MINT^2^	–				−0.153**	0.910		
pH	0.242**	0.810			–			
pH^2^	–				–			

Note

Model sets involved all possible combinations of explanatory variables, for two groups of variables: Group 1: minimum temperature of the coldest month (MINT), mean annual precipitation (MAP). Group 2: MINT, soil pH (pH). Coefficients for the model with the lowest AIC_c_ for a given variable group are shown. The Akaike weight for each variable is based on the full model set per group. The superscript 2 indicates the quadratic form of the variables. Pseudo‐*r* ^2^, explained the variance of the SAR model. Moran's *I*, measure of residual spatial autocorrelation.

Significance levels:

*p* < 0.001;

a *p* < 0.01;

b *p* < 0.05. *ns*, not significant.

**Figure 4:**
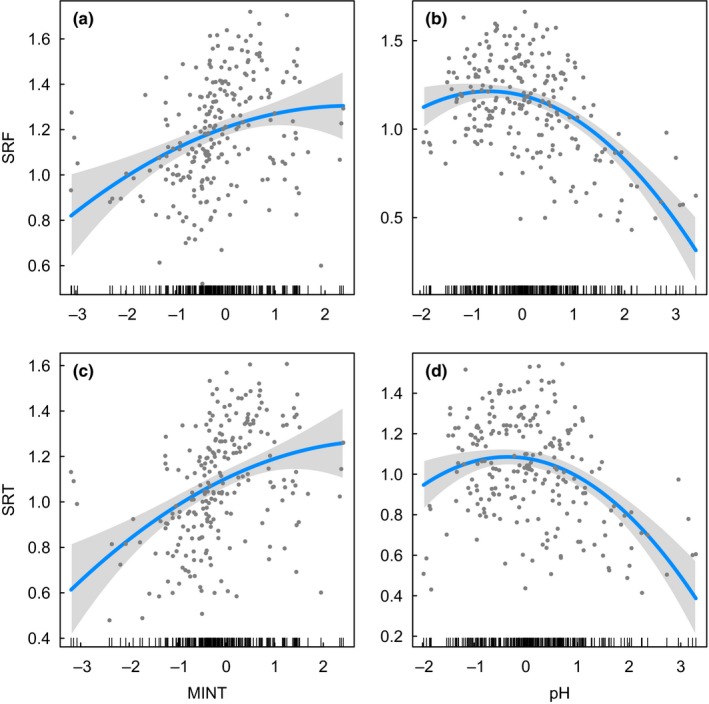
Relationship between species richness and minimum temperature of the coldest month (MINT) or soil pH (pH) in the ordinary least squares (OLS) regression models. SRF indicates species richness at family level (i.e., Theaceae), and SRT indicates species richness at tribe level (i.e., Theeae). Species richness responses to each predictor in multiple models are shown one at a time, holding all other predictors constant. The regression line is given in blue, and the 95% confidence interval is given in gray. Multimodel inference results are given in Supporting Information

**Figure 5:**
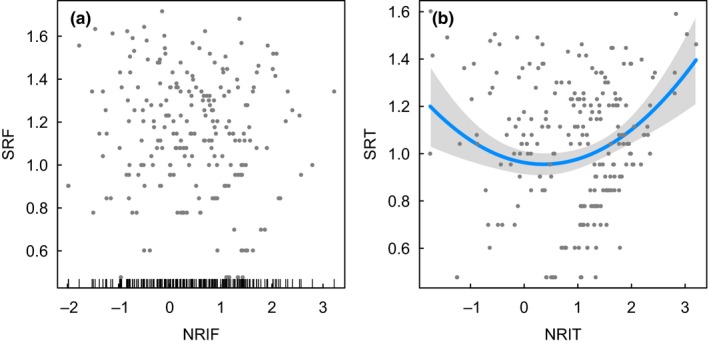
Relationship between species richness and net relatedness index (NRI) based on ordinary least squares (OLS) regression models. SRF and NRIF indicate species richness and NRI at family level (i.e., Theaceae), SRT and NRIT indicate species richness and NRI at tribe level (i.e., Theeae). The regression line is given in blue, and the 95% confidence interval is given in gray. The scatter plot, with no significant relationship, is also shown. Multimodel inference results are given in Supporting Information

**Figure 6:**
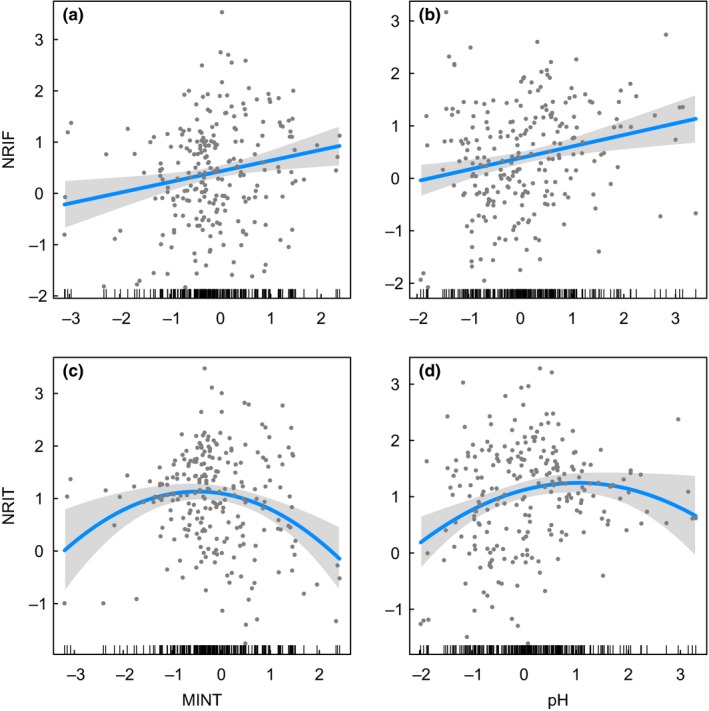
Relationship between net relatedness index (NRI) and minimum temperature of the coldest month (MINT) or soil pH (pH) based on ordinary least squares (OLS) regression models. NRIF indicates NRI at family level (i.e., Theaceae), and NRIT indicates NRI at tribe level (i.e., Theeae). The NRI responses to each predictor in multiple models are shown one at a time, holding all other predictors constant. The regression line is given in blue, and the 95% confidence interval is given in gray. Multimodel inference results are given in Supporting Information

The OLS models provided consistent results with the SAR analyses for the relationships between species richness and soil pH (Tables 3 and Supporting Information Table S2, Figure 4), as well as the relationships between species richness and phylogenetic structure (NRI) (Tables 4 and Supporting Information Table S3, Figure 5). The main differences identified was that the effect of pH on NRI at tribe level was significant in the OLS modeling, but not supported in the SAR analyses (Tables 5 and Supporting Information Table S4, Figure 6). However, some differences for less supported environmental factors were also found (Tables 3, and 5, Supporting Information Tables S2, and S4, Figures S2 and S3).

## 4 DISCUSSION

We here compiled species distribution data, environmental data, and phylogeny to assess the drivers of species richness in a key subtropical woody plant family (Theaceae) across China. Both the phylogenetic relationship and the divergence times estimated in our results were consistent with Yu et al. (2017). Our results indicate that both environmental and evolutionary factors play important roles in shaping species richness patterns of tea family species across China. The strong relationship between soil pH and species richness was found consistently at both family level and tribe level, supporting the idea that environmental factors explain much of the variation in the species richness of Theaceae. Although the species richness patterns along environmental gradients at family and tribe levels were similar, the processes for these patterns differed when considering evolutionary dynamics.

Species richness patterns for the whole tea family as well as the Theeae tribe in China exhibited significant relationships with environmental variables, of which soil pH was the strongest predictive variable, having a negative correlation with species richness in Theaceae and in Theeae (Table 3). Generally, climatic variables have been considered the main environmental factors that control large‐scale patterns of species richness (Francis & Currie, 2003; McGill, 2010). At small scales, edaphic properties, like soil pH, have been shown to be key environmental factors influencing plant species richness patterns (Dubuis et al., 2013; Zellweger et al., 2016). However, our results indicate that soil is the most important environmental factor influencing the richness patterns of Theaceae, even on a biogeographic scale. The large areas of red soil in the subtropical region across south China used in our study, combined with the special adaptations of the tea family for low pH soils may explain this pattern. Theaceae's tolerance of low pH has been illustrated through cultivation studies of tea plants (*Camellia sinensis*), which have shown them to grow well in soils of low pH (appropriate 4.0–5.5) and a high Al concentration (Fu, Wang, & Ding, 2013). Notably, a high Al concentration has been found to stimulate tea plant growth (Konishi, Miyamoto, & Taki, 1985), but the same treatment would be toxic for most other plants (Fung & Wong, 2002).

The analysis of the whole family showed a strong positive relationship between NRI and pH (Table 5), which combined with the negative relationship between species richness and pH, provides support to the time and niche conservatism effect. However, no relationship was found between NRI and species richness (Table 4). This lacking species richness–NRI relationship may have resulted from the canceling out of different underlying relationships, caused by the following different processes: The processes of the time‐for‐speciation hypothesis may lead to an assemblage with many species and low NRI (Qian et al., 2015; Stephens & Wiens, 2003), whereas the processes of the diversification rates hypothesis could also lead to many species, but with high NRI (Qian et al., 2015).

Our results showed that there is a significant relationship between species richness and NRI for the Theeae tribe (Table 4), but the U‐shaped relationship is not consistent with either the pure time‐for‐speciation hypothesis or the diversification rate hypothesis. Some species‐rich areas (species richness >= 25; Figure 3b) exhibited phylogenetically clustered patterns, while others tended to be phylogenetically overdispersed (Figure 3d). The species compositions of these two types of species‐rich areas mainly included the *Camellia*, the *Pyrenaria* or the *Polyspora*. However, the proportions of *Pyrenaria* and/or *Polyspora* species (>19%) in areas with overdispersed phylogenetic structure were higher than in areas where phylogenetic structures were clustering (<17%). According to the locations of these species‐rich areas, most of them were situated in one of three areas: the southwestern region bordering Vietnam, the Nanling Mountains and surrounding land in southcentral China, and the northeastern edge of the Yungui Plateau. In previous studies of endemic Chinese plants, these areas have been hypothesized to be former glacial refuges (Huang et al., 2011, 2015 ; López‐Pujol, Zhang, Sun, Ying, & Ge, 2011). Hence, we suggest that the long‐term stable environmental conditions in these refuge areas may have enabled them to harbor more species from clades that are distantly related via relictual survival, while others may primarily have harbored one clade, due to in situ diversification within the area. The presence of the above processes in species‐rich areas should lead to U‐shaped relationship.

Our results show that processes underlying species richness patterns for tea family species in China differ between taxonomic levels. We suggest this might be because richness patterns at the family level are the sum of processes occurring within different clades. This would even out clade‐specific idiosyncratic evolutionary patterns, whereas tribe‐level patterns would reflect a tribe's specific evolutionary history and response to environmental factors (Bregovic & Zagmajster, 2016; Terribile, Olalla‐Tarraga, Diniz, & Rodriguez, 2009). For example, *Stewartia*, which is the sole genus in Stewartieae, has both deciduous and evergreen species in China, whereas all species across China in Gordonieae and Theeae are evergreen (Li, 2011; Li, Li, Tredici, Corajod, & Fu, 2013). Because Gordonieae and Stewartieae contributed considerably to the overall phylogenetic structure pattern found in our study, the phylogenetic structure's responses to environmental factors changed after removing these two clades (Table 5). However, given their low richness, they contributed little to the overall pattern of tea family species richness. Therefore, the species richness–environmental relationships were similar at the family‐wide and the tribe level (Table 3).

In conclusion, soil pH provides the strongest explanatory predictor for the geographic variation in species richness of Theaceae across China. This is likely linked to the Theaceae family's specific adaptations to acidic soil. This pattern contrasts the general assumption that soil only influences ecological patterns at small scales (Palpurina et al., 2017; Pärtel, 2002). Furthermore, the relationships between species richness and phylogenetic structure caused by different processes (time‐for‐speciation vs. diversification rate) may be canceling each other out at the family level, leading to no species richness–NRI relation at this taxonomic level. At the tribe level, the relationship between species richness and phylogenetic structure was significant, but more complex than predicted by time‐for‐speciation and diversification rate hypotheses. Some species‐rich areas tended to host relatively distantly related species of Theeae, while others exhibited phylogenetically clustered patterns. This is likely due to different refuges in southern China (López‐Pujol et al., 2011) having played different roles in tea species’ diversity. Some likely allowed several deep lineages to survive, while others promoted the diversification of a single lineage. In addition, our results imply that the forces shaping species richness patterns vary among different groups and with taxonomic scale, even within the same family. Overall, our findings show that environmental and evolutionary processes interact in complex ways to shape species richness patterns within the subtropical forest biome.

## CONFLICT OF INTEREST

None declared.

## AUTHOR CONTRIBUTIONS

XCM, JTZ, JCS, and MDR conceived the ideas. MDR, JCS, and MJS designed the methodology. KPM and MGZ collected the data. MDR and XGX analyzed the data. All authors contributed to the manuscript text.

## DATA ACCESSIBILITY

Data available from the Dryad Digital Repository: https://doi.org/10.5061/dryad.s526b27.
