# Supplementary material for: Environmental and evolutionary drivers of diversity patterns in the tea family (Theaceae s.s.) across China
Source: Ecol Evol. 2018 Nov 8;8(23):11663–76. doi: 10.1002/ece3.4619 (PMC6303774; doi:10.1002/ece3.4619)
Supplement: Supplementary file 1 [file ECE3-8-11663-s001.pdf]

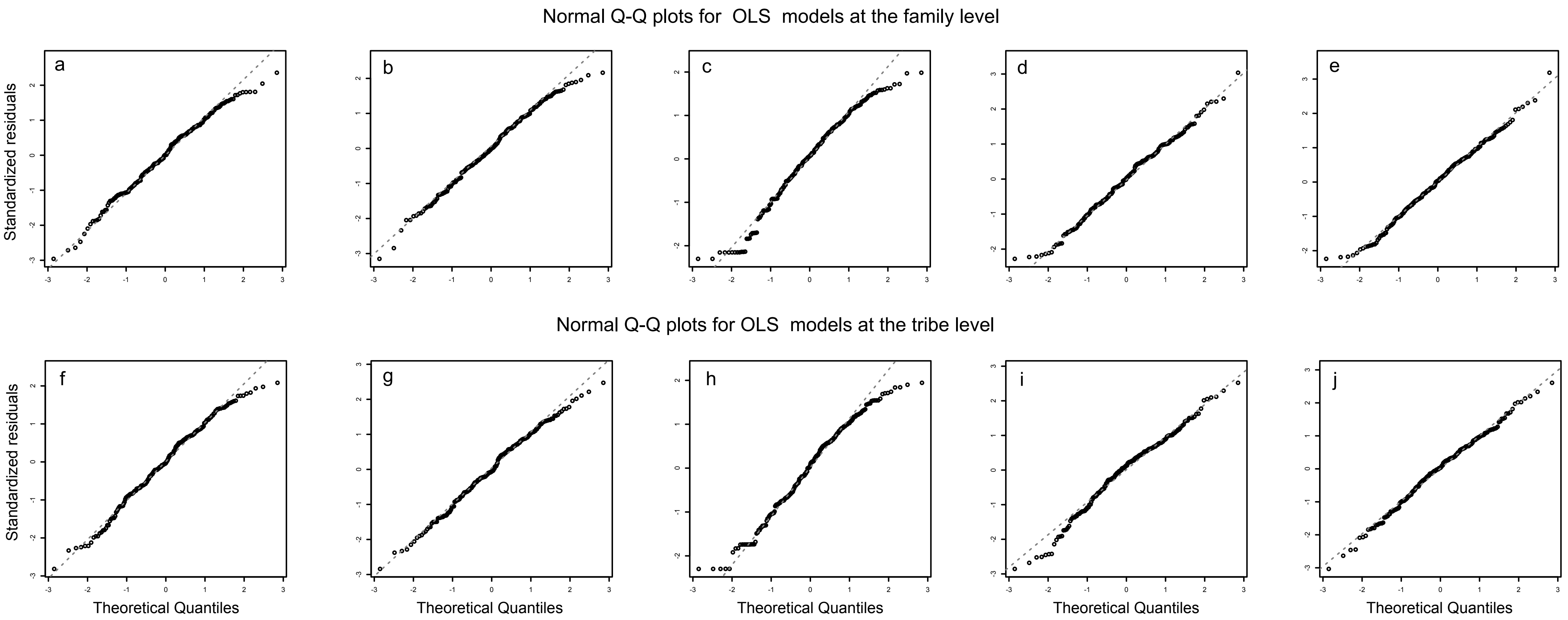
Normal Q-Q plots for OLS models at the family level
Normal Q-Q plots for OLS models at the tribe level
Standardized residuals
Standardized residuals
Theoretical Quantiles
Theoretical Quantiles
Theoretical Quantiles
Theoretical Quantiles
Theoretical Quantiles
a
b
c
d
e
f
g
h
i
j
