# Supplementary figures and images for: Environmental and evolutionary drivers of diversity patterns in the tea family (Theaceae s.s.) across China
Source: Ecol Evol. 2018 Nov 8;8(23):11663–76. doi: 10.1002/ece3.4619 (PMC6303774; doi:10.1002/ece3.4619)

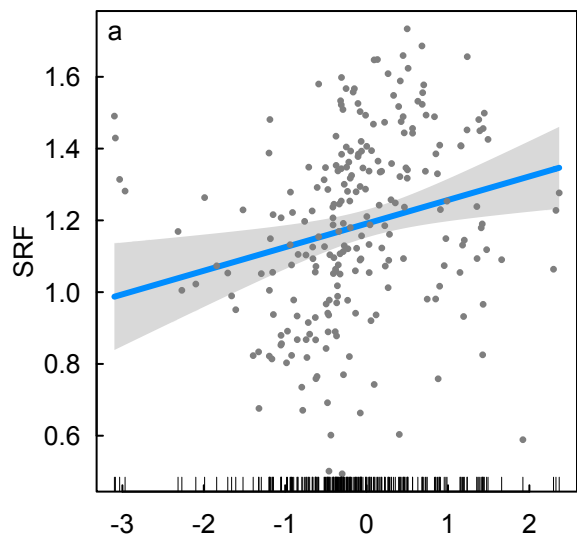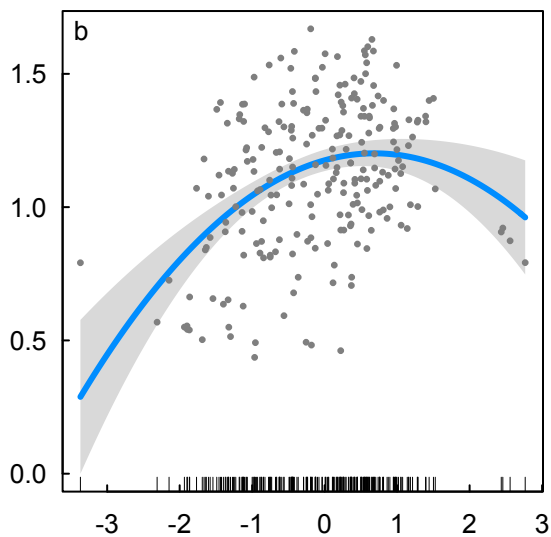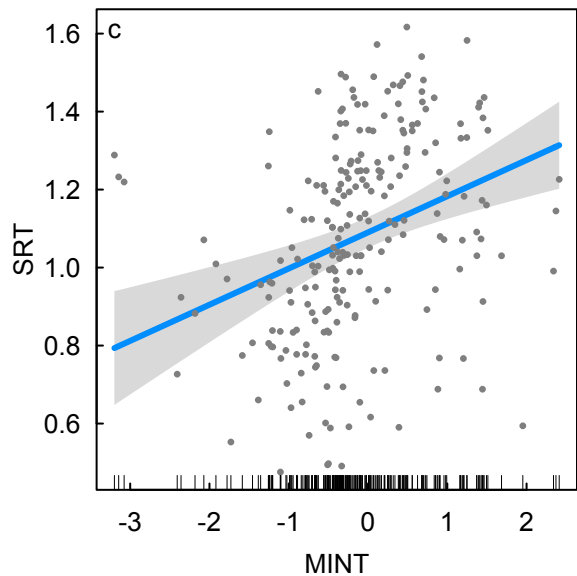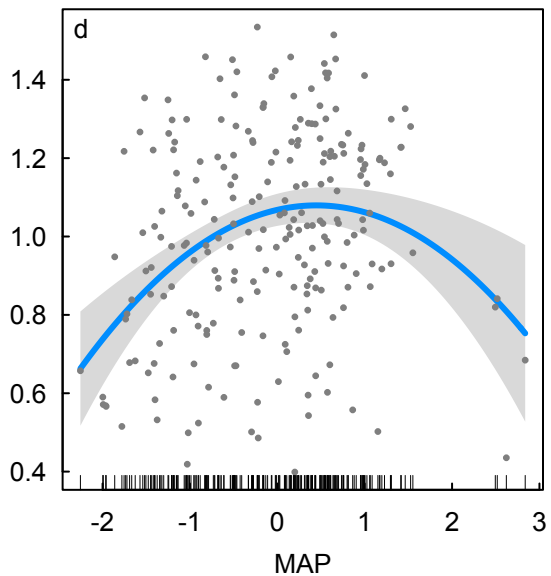

Supplement: Supplementary file 2 [file ECE3-8-11663-s002.pdf]

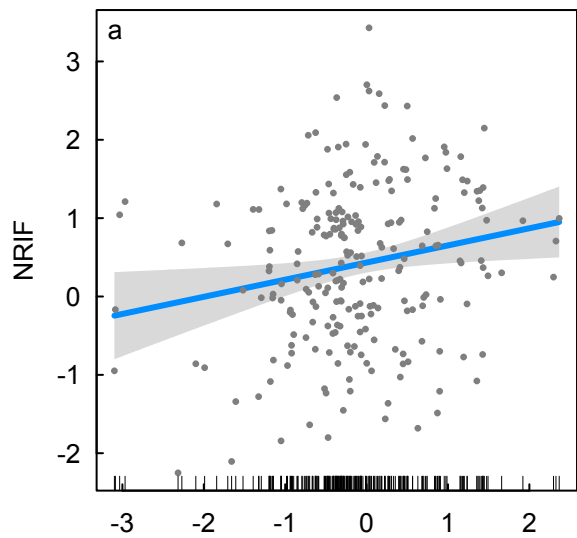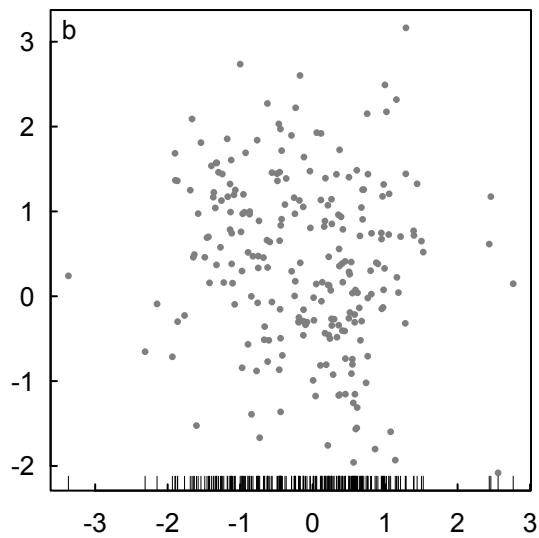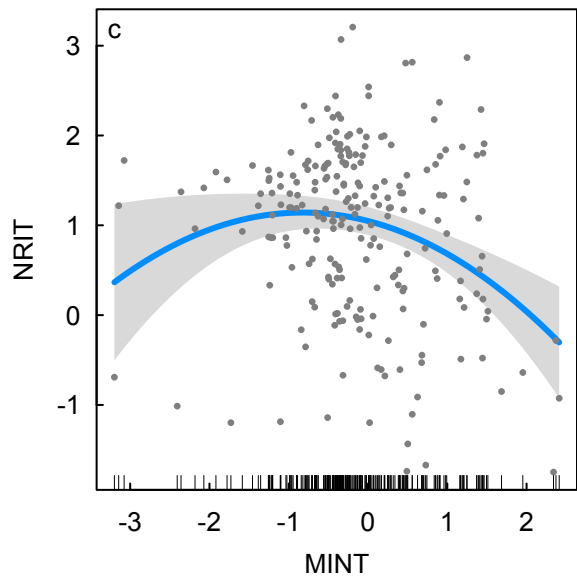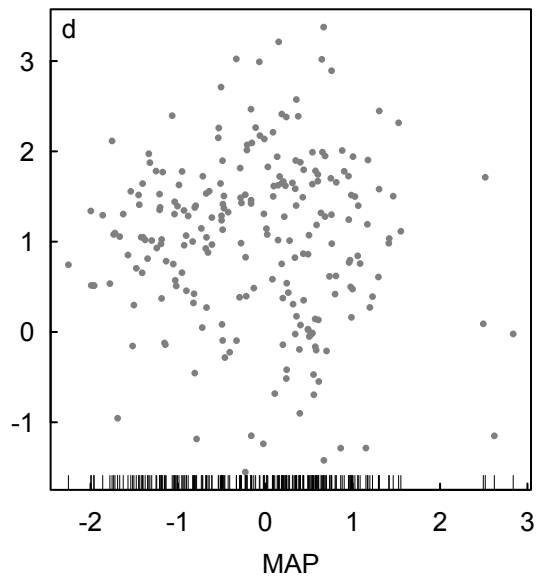

Supplement: Supplementary file 3 [file ECE3-8-11663-s003.pdf]
