# Supplementary material for: Environmental and evolutionary drivers of diversity patterns in the tea family (Theaceae s.s.) across China
Source: Ecol Evol. 2018 Nov 8;8(23):11663–76. doi: 10.1002/ece3.4619 (PMC6303774; doi:10.1002/ece3.4619)
Supplement: Supplementary file 4 [file ECE3-8-11663-s004.docx]

Table S1 Accession numbers for the Theaceae s.s. and outgroup species (the last 10) used in this study.

| Taxon | *atpI*-*H* | *atpB*-*rbcL* | ITS | *matK* | *matK*_*trnK*-*psbA* | *rbcL*-*accD* | *rpl32*-*trnL* | *psbA*-*trnH* | *rbcL* | *rpl16* | *trnL*-*trnF* |
| --- | --- | --- | --- | --- | --- | --- | --- | --- | --- | --- | --- |
| *Apterosperma oblata* | KJ198033 | HQ158556 | AY070324 | KJ197940 | KJ198002 | - | - | - | KJ197971 | AY070305 | HQ158592 |
| *Camellia amplexicaulis* | AB364664 | - | EU579676 | - | - | - | - | - | - | - | - |
| *Camellia anlungensis* | KJ198013 | - | FJ432095 | KJ197920 | KJ197982 | - | - | - | KJ197951 | - | - |
| *Camellia azalea* | - | - | EU579681 | - | - | - | - | - | - | - | - |
| *Camellia brevistyla* | AB364670 | HQ158531 | EU579683 | - | - | - | - | - | - | - | HQ158568 |
| *Camellia caudata* | AB443617 | HQ158548 | EU579684 | - | - | AB207870 | - | - | - | - | HQ158585 |
| *Camellia chekiangoleosa* | AB364655 | - | FJ432101 | HQ427374 | - | - | - | HQ427072 | HQ427229 | - | - |
| *Camellia chrysanthoides* | - | HQ158552 | AF315483 | - | - | - | GQ487396 | GQ487336 | - | GQ487366 | HQ158589 |
| *Camellia cordifolia* | - | - | EU579689 | - | - | - | - | - | - | - | - |
| *Camellia costata* | - | - | - | - | - | - | - | - | - | KF156834 | - |
| *Camellia costei* | - | - | EU579691 | - | - | - | - | - | - | - | - |
| *Camellia crapnelliana* | AB443611 | HQ158536 | EU579692 | - | - | - | - | - | - | - | HQ158573 |
| *Camellia crassicolumna* | KJ198019 | - | EU579679 | KJ197926 | KJ197988 | - | - | - | KJ197957 | - | - |
| *Camellia crassiphylla* | - | - | - | - | - | - | - | GQ487337 | - | GQ487367 | GQ487427 |
| *Camellia cuspidata* | - | - | EU579693 | HQ427370 | - | AB207871 | - | HQ427068 | HQ427225 | - | - |
| *Camellia drupifera* | AB364676 | - | FJ432126 | - | - | - | - | - | - | - | - |
| *Camellia edithae* | KJ198025 | - | EU579696 | KJ197932 | KJ197994 | - | - | - | KJ197963 | - | - |
| *Camellia elongata* | - | - | - | - | - | - | GQ487398 | GQ487338 | - | GQ487368 | GQ487428 |
| *Camellia euphlebia* | - | - | EU579697 | - | - | - | GQ487399 | GQ487339 | - | GQ487369 | GQ487429 |
| *Camellia euryoides* | - | - | FJ432102 | - | - | - | - | - | - | - | - |
| *Camellia fascicularis* | - | - | AF315485 | - | - | - | - | - | - | - | - |
| *Camellia flava* | - | - | EU579699 | - | - | - | GQ487400 | GQ487340 | - | GQ487370 | GQ487430 |
| *Camellia flavida* | - | - | AF315480 | - | - | - | GQ487401 | GQ487341 | - | GQ487371 | GQ487431 |
| *Camellia fluviatilis* | - | HQ158544 | FJ432103 | - | - | - | - | - | - | - | HQ158581 |
| *Camellia formosensis* | - | - | EF544719 | - | - | - | - | - | - | - | - |
| *Camellia forrestii* | - | - | EU579703 | - | - | - | GQ487402 | GQ487342 | - | GQ487372 | GQ487432 |
| *Camellia fraterna* | - | - | EU579705 | - | - | - | - | HQ427067 | HQ427224 | - | - |
| *Camellia furfuracea* | AB443614 | HQ158549 | EU579706 | - | - | AB207872 | GQ487403 | GQ487343 | - | GQ487373 | HQ158586 |
| *Camellia gilbertii* | - | - | EU579709 | - | - | - | - | - | - | - | - |
| *Camellia grandibracteata* | KJ198029 | - | - | KJ197936 | KJ197998 | - | - | - | KJ197967 | - | - |
| *Camellia granthamiana* | - | - | EU579710 | AF380073 | - | - | - | - | AF380034 | - | HQ158584 |
| *Camellia grijsii* | AB364673 | HQ158550 | EU579795 | - | - | - | - | - | - | - | HQ158587 |
| *Camellia gymnogyna* | - | - | - | - | - | - | GQ487404 | - | - | GQ487374 | GQ487434 |
| *Camellia hekouensis* | - | - | - | - | - | - | GQ487405 | GQ487345 | - | GQ487375 | GQ487435 |
| *Camellia hongkongensis* | KJ198024 | - | EU579717 | KJ197931 | KJ197993 | AB207873 | - | - | KJ197962 | - | - |
| *Camellia huana* | KJ198015 | - | AF315490 | KJ197922 | KJ197984 | - | - | - | KJ197953 | - | - |
| *Camellia impressinervis* | - | HQ158553 | EU579721 | - | - | - | GQ487406 | GQ487346 | - | GQ487376 | HQ158590 |
| *Camellia indochinensis* | KJ198023 | - | AF315479 | KJ197930 | KJ197992 | - | GQ487409 | GQ487349 | KJ197961 | GQ487379 | GQ487439 |
| *Camellia japonica* | AB364653 | - | EU579723 | AF380074 | - | AB207875 | - | - | AF380035 | - | AF396226 |
| *Camellia kissii* | AB364675 | HQ158524 | EU579726 | - | - | AB207876 | - | - | - | - | HQ158561 |
| *Camellia kwangsiensis* | - | - | FJ432106 | - | - | - | GQ487407 | GQ487347 | - | GQ487377 | GQ487437 |
| *Camellia lanceolata* | - | - | - | AF380075 | - | - | - | - | - | - | - |
| *Camellia lawii* | - | - | EU579730 | - | - | - | GQ487408 | GQ487348 | - | GQ487378 | GQ487438 |
| *Camellia leptophylla* | - | - | EU579731 | KJ806275 | - | - | - | - | KJ806275 | KJ806275 | - |
| *Camellia longipedicellata* | - | - | EU579734 | - | - | - | GQ487410 | GQ487350 | - | GQ487380 | GQ487440 |
| *Camellia longissima* | - | - | - | - | - | - | GQ487411 | GQ487351 | - | GQ487381 | GQ487441 |
| *Camellia lutchuensis* | AB364668 | - | EU579783 | - | - | - | - | - | - | - | - |
| *Camellia mairei* | KJ198026 | - | FJ432108 | KJ197933 | KJ197995 | - | - | - | KJ197964 | - | - |
| *Camellia micrantha* | - | HQ158528 | AF315484 | - | - | - | GQ487412 | GQ487352 | - | GQ487382 | HQ158565 |
| *Camellia murauchii* | - | - | - | - | - | - | GQ487413 | GQ487353 | - | GQ487383 | GQ487443 |
| *Camellia oleifera* | AB364666 | - | FJ432111 | - | - | - | GQ487415 | GQ487355 | GQ436646 | GQ487385 | HQ158562 |
| *Camellia pachyandra* | - | - | EU579748 | - | - | - | - | - | - | - | - |
| *Camellia parvimuricata* | KJ198012 | - | FJ432112 | KJ197919 | KJ197981 | - | - | - | KJ197950 | - | - |
| *Camellia petelotii* | AB364656 | HQ158522 | EU579753 | - | - | - | GQ487414 | GQ487354 | - | GQ487384 | HQ158559 |
| *Camellia pingguoensis* | KJ198016 | - | AF315475 | KJ197923 | KJ197985 | - | - | - | KJ197954 | - | - |
| *Camellia piquetiana* | - | - | EU579757 | - | - | - | - | - | - | - | - |
| *Camellia pitardii* | AB443616 | - | FJ432115 | - | - | AB207877 | - | - | - | - | - |
| *Camellia polyodonta* | AB443618 | - | EU579760 | - | - | - | - | - | - | - | HQ158583 |
| *Camellia ptilophylla* | - | - | FJ432116 | - | - | - | - | - | - | - | - |
| *Camellia pubifurfuracea* | - | - | EU579761 | - | - | - | - | - | - | - | - |
| *Camellia pubipetala* | - | - | - | - | - | - | GQ487417 | GQ487357 | - | GQ487387 | GQ487447 |
| *Camellia pyxidiacea* | KJ198022 | - | FJ432118 | KJ197929 | KJ197991 | - | - | - | KJ197960 | - | - |
| *Camellia reticulata* | - | - | HM061380 | - | - | - | GQ487418 | GQ487358 | - | GQ487388 | GQ487448 |
| *Camellia rhytidocarpa* | KJ198021 | - | EU579763 | KJ197928 | KJ197990 | - | - | - | KJ197959 | - | - |
| *Camellia salicifolia* | - | HQ158535 | EU579765 | - | - | AB207878 | - | - | - | - | HQ158572 |
| *Camellia saluenensis* | AB364671 | - | EU579767 | - | - | AB207879 | - | - | - | - | - |
| *Camellia semiserrata* | - | - | EU579770 | KJ197925 | KJ197987 | - | - | - | - | - | - |
| *Camellia sinensis* | - | HQ158551 | FJ432121 | AF380077 | - | - | GQ487419 | GQ487359 | AF380037 | GQ487389 | GQ487449 |
| *Camellia subintegra* | KJ198017 | - | EU579776 | KJ197924 | KJ197986 | - | - | - | KJ197955 | - | - |
| *Camellia synaptica* | - | - | - | KJ197921 | KJ197983 | - | - | - | KJ197952 | - | - |
| *Camellia szechuanensis* | - | - | EU579777 | - | - | - | - | - | - | - | - |
| *Camellia tachangensis* | KJ198028 | - | EU579725 | KJ197935 | KJ197997 | - | - | - | KJ197966 | - | - |
| *Camellia taliensis* | KJ198027 | - | FJ432122 | KJ197934 | KJ197996 | AB207881 | JX161633 | - | KJ197965 | - | - |
| *Camellia transarisanensis* | - | - | EU579782 | - | - | - | - | - | - | - | - |
| *Camellia tsaii* | - | - | EU579784 | - | - | AB207882 | - | - | - | - | - |
| *Camellia tuberculata* | - | - | EU579688 | - | - | - | - | - | - | - | - |
| *Camellia wardii* | - | - | - | - | - | - | GQ487422 | GQ487362 | - | GQ487392 | GQ487452 |
| *Camellia yunnanensis* | - | - | EU579796 | - | - | - | GQ487423 | GQ487363 | - | GQ487393 | GQ487453 |
| *Franklinia alatamaha* | - | - | HM100445 | AF380082 | - | - | - | HM100514 | AF380040 | - | HM100590 |
| *Gordonia brandegeei* | - | - | AY070325 | AF380084 | - | - | - | - | - | AY070306 | - |
| *Gordonia lasianthus* | - | - | HM100446 | AF380085 | - | - | - | HM100515 | AF380042 | - | HM100591 |
| *Laplacea portoricensis* | - | - | - | AF380089 | - | - | - | - | AF380046 | - | - |
| *Polyspora axillaris* | - | - | AY214930 | AF380090 | - | - | - | - | AF380047 | - | AY214937 |
| *Polyspora chrysandra* | - | - | AY214931 | AF380091 | - | - | - | - | AF380048 | - | AF534678 |
| *Polyspora hainanensis* | KJ198031 | - | AY214932 | KJ197938 | KJ198000 | - | - | - | KJ197969 | - | AY216566 |
| *Polyspora longicarpa* | KJ198030 | - | AF456264 | KJ197937 | KJ197999 | - | - | - | KJ197968 | - | AY214938 |
| *Polyspora speciosa* | KJ198032 | - | - | AF380093 | KJ198001 | - | - | - | AF380050 | - | - |
| *Pyrenaria diospyricarpa* | - | - | AF456270 | - | - | - | - | - | - | - | - |
| *Pyrenaria hirta* | KJ198034 | - | - | KJ197941 | KJ198003 | - | - | - | KJ197972 | - | AY216578 |
| *Pyrenaria kwangsiensis* | KJ198040 | - | AF456265 | KJ197947 | KJ198009 | - | - | - | KJ197978 | - | AY216570 |
| *Pyrenaria maculatoclada* | - | - | AF456276 | - | - | - | - | - | - | - | AY216573 |
| *Pyrenaria menglaensis* | - | - | EU579798 | - | - | - | - | - | - | - | - |
| *Pyrenaria microcarpa* | KJ198041 | HQ158529 | AF456277 | KJ197948 | KJ198010 | - | - | HQ427074 | KJ197979 | - | AY216571 |
| *Pyrenaria oblongicarpa* | - | - | AF456267 | - | - | - | GQ487424 | GQ487364 | - | GQ487394 | GQ487454 |
| *Pyrenaria pingpienensis* | - | - | AF456278 | - | - | - | - | - | - | - | AY216572 |
| *Pyrenaria sophiae* | - | - | AF456279 | - | - | - | - | - | - | - | AF499816 |
| *Pyrenaria spectabilis* | KJ198037 | - | AF456280 | KJ197946 | KJ198008 | - | GQ487425 | GQ487365 | KJ197977 | GQ487395 | GQ487455 |
| *Pyrenaria wuana* | - | - | AF456281 | - | - | - | - | - | - | - | AY216574 |
| *Schima argentea* | - | - | HM100438 | AF380096 | - | - | - | HM100536 | AF380053 | - | HM100604 |
| *Schima khasiana* | - | - | HM100439 | - | - | - | - | HM100538 | - | - | HM100605 |
| *Schima noronhae* | - | - | - | AF380097 | - | - | - | - | AF380054 | - | - |
| *Schima remotiserrata* | - | - | HM100440 | AF380098 | - | - | - | HM100539 | AF380055 | - | - |
| *Schima sericans* | - | - | HM100441 | - | - | - | - | HM100540 | - | - | HM100608 |
| *Schima sinensis* | - | - | AY096020 | - | - | - | - | - | - | - | AF499811 |
| *Schima superba* | - | HQ158534 | HM100442 | AF380099 | - | - | - | HM100541 | AF421103 | - | HM100609 |
| *Schima wallichii* | - | - | HM100444 | AF380100 | - | - | - | HM100543 | AF380056 | - | HM100611 |
| *Stewartia calcicola* | - | - | AY070311 | - | - | - | - | - | - | AY070293 | - |
| *Stewartia cordifolia* | - | - | HM100447 | - | - | - | - | HM100516 | - | - | HM100592 |
| *Stewartia crassifolia* | - | - | HM100449 | - | - | - | - | HM100518 | - | - | HM100593 |
| *Stewartia laotica* | - | - | HM100450 | - | - | - | - | HM100520 | - | - | HM100594 |
| *Stewartia malacodendron* | - | - | HM100480 | AF380101 | - | - | - | HM100558 | AF380057 | AY070295 | HM100623 |
| *Stewartia micrantha* | - | - | HM100452 | - | - | - | - | HM100521 | - | - | - |
| *Stewartia monadelpha* | - | - | HM100485 | - | - | - | - | HM100563 | - | - | HM100628 |
| *Stewartia obovata* | - | - | HM100455 | - | - | - | - | HM100524 | - | - | - |
| *Stewartia ovata* | - | - | HM100488 | AF380103 | - | - | - | HM100567 | - | AY070299 | HM100632 |
| *Stewartia pteropetiolata* | - | HQ158523 | HM100458 | AF380087 | - | - | - | HM100528 | AF380044 | - | HM100596 |
| *Stewartia rostrata* | - | - | HM100498 | - | - | - | - | HM100576 | - | AY070302 | HM100642 |
| *Stewartia rubiginosa* | - | - | HM100505 | - | - | - | - | HM100582 | - | - | HM100646 |
| *Stewartia sichuanensis* | - | - | HM100457 | - | - | - | - | HM100526 | - | - | - |
| *Stewartia sinensis* | KJ198036 | - | HM100470 | AF380106 | KJ198005 | - | - | HM100555 | KJ197974 | AY070304 | HM100615 |
| *Stewartia sinii* | - | - | HM100461 | - | - | - | - | HM100531 | - | - | HM100599 |
| *Stewartia tonkinensis* | - | - | HM100463 | - | - | - | - | HM100533 | - | - | HM100601 |
| *Stewartia villosa* | - | - | HM100464 | AF380086 | - | - | - | HM100534 | AF380043 | AY070289 | HM100602 |
| *Alniphyllum fortunei* | - | - | AF396437 | KJ510930 | - | - | - | KP095282 | KP095009 | - | AF396162 |
| *Pterostyrax psilophyllus* | - | DQ317980 | AF396447 | - | - | - | - | DQ317988 | AF396156 | - | AF396184 |
| *Styrax obassia* | - | - | AF327479 | - | - | - | - | - | AF396158 | - | AB237440 |
| *Styrax officinalis* | - | - | AF327489 | AJ429300 | - | - | - | - | AF396159 | EU863153 | AF396198 |
| *Symplocos adenopus* | - | - | AY336332 | HQ415340 | - | - | - | KP095264 | KP095093 | AY336424 | AY336487 |
| *Symplocos anomala* | - | - | AY336291 | AY679808 | - | - | - | HQ427076 | HQ427233 | KF002821 | AY336453 |
| *Symplocos congesta* | - | - | AY336311 | AY679810 | - | - | - | KJ687431 | KJ688840 | KF002823 | AY630745 |
| *Symplocos paniculata* | - | - | AY336263 | AY336340 | - | - | - | HQ427077 | HQ427234 | AY336382 | AY336430 |
| *Symplocos sumuntia* | - | - | AY336321 | AY336372 | - | - | - | HQ427075 | HQ427232 | AY336417 | AY336476 |
| *Symplocos wikstroemiifolia* | - | - | AY336273 | AY336346 | - | - | - | HQ415517 | KP094902 | AY336390 | AY336436 |
