# Supplementary material for: Environmental and evolutionary drivers of diversity patterns in the tea family (Theaceae s.s.) across China
Source: Ecol Evol. 2018 Nov 8;8(23):11663–76. doi: 10.1002/ece3.4619 (PMC6303774; doi:10.1002/ece3.4619)
Supplement: Supplementary file 5 [file ECE3-8-11663-s005.docx]

Table S2 Multimodel inference from the ordinary least squares (OLS) regression models of species richness against environmental predictors for Theaceae and Theeae.

|  | Theaceae |  |  |  |  | Theeae |  |  |  |
| --- | --- | --- | --- | --- | --- | --- | --- | --- | --- |
| Model  Parameters | Coefficients | Akaike  weight | OLS *r*^2^ | Moran’s *I* |  | Coefficients | Akaike  weight | OLS *r*^2^ | Moran’s *I* |
| Group 1 |  |  |  |  |  |  |  |  |  |
|  |  |  | 0.276 | 0.359** |  |  |  | 0.262 | 0.335** |
| MINT | 0.066** | 0.95 |  |  |  | 0.093*** | 1.00 |  |  |
| MINT^2^ | - |  |  |  |  | - |  |  |  |
| MAP | 0.076*** | 1.00 |  |  |  | 0.051* | 0.87 |  |  |
| MAP^2^ | -0.056*** | 1.00 |  |  |  | -0.057*** | 1.00 |  |  |
| Group 2 |  |  |  |  |  |  |  |  |  |
|  |  |  | 0.393 | 0.303** |  |  |  | 0.329 | 0.297** |
| MINT | 0.077*** | 1.00 |  |  |  | 0.103*** | 1.00 |  |  |
| MINT^2^ | -0.016n.s. | 0.53 |  |  |  | -0.016n.s. | 0.52 |  |  |
| pH | -0.072*** | 1.00 |  |  |  | -0.037* | 0.72 |  |  |
| pH^2^ | -0.055*** | 1.00 |  |  |  | -0.053*** | 1.00 |  |  |

Model sets involved all possible combinations of explanatory variables, for two groups of variables: Group 1: minimum temperature of the coldest month (MINT), mean annual precipitation (MAP). Group 2: MINT, soil pH (pH). Coefficients for the model with the lowest AICc for a given variable group are shown. The Akaike weight for each variable is based on the full model set per group. The superscript 2 indicates the quadratic form of the variable. OLS *r*^2^, the explained variance of the OLS regression model. Moran’s *I*, measure of residual spatial autocorrelation.

Significance levels: ****P* < 0.001; ***P* < 0.01; **P* < 0.05. n.s., not significant.
