# Supplementary material for: Environmental and evolutionary drivers of diversity patterns in the tea family (Theaceae s.s.) across China
Source: Ecol Evol. 2018 Nov 8;8(23):11663–76. doi: 10.1002/ece3.4619 (PMC6303774; doi:10.1002/ece3.4619)
Supplement: Supplementary file 6 [file ECE3-8-11663-s006.docx]

Table S3 Multimodel inference from the ordinary least squares (OLS) regression models of species richness against phylogenetic predictors for Theaceae and Theeae.

|  | Theaceae |  |  |  |  | Theeae |  |  |  |
| --- | --- | --- | --- | --- | --- | --- | --- | --- | --- |
| Model  Parameters | Coefficients | Akaike  weight | OLS *r*^2^ | Moran’s *I* |  | Coefficients | Akaike  weight | OLS *r*^2^ | Moran’s *I* |
|  |  |  | 0.000 | 0.526** |  |  |  | 0.057 | 0.544** |
| NRI | -0.020n.s. | 0.36 |  |  |  | -0.040n.s. | 0.52 |  |  |
| NRI^2^ | - |  |  |  |  | 0.055*** | 1.00 |  |  |

Coefficients for the model with lowest AICc for a give variable group are shown. The Akaike weight for each variable based on the full model sets per group. The superscript 2 indicates the quadratic form of the variable. OLS *r*^2^, the explained variance of the OLS regression model. Moran’s *I*, measure of residual spatial autocorrelation.

Significance levels: ****P* < 0.001; ***P* < 0.01; **P* < 0.05. n.s., not significant.
