# Supplementary material for: Environmental and evolutionary drivers of diversity patterns in the tea family (Theaceae s.s.) across China
Source: Ecol Evol. 2018 Nov 8;8(23):11663–76. doi: 10.1002/ece3.4619 (PMC6303774; doi:10.1002/ece3.4619)
Supplement: Supplementary file 8 [file ECE3-8-11663-s008.docx]

**Supporting Information**

Table S1 Accession numbers for the Theaceae s.s. and outgroup species used in this study.

Table S2 Multimodel inference from the ordinary least squares (OLS) regression models of species richness against environmental predictors for Theaceae and Theeae.

Table S3 Multimodel inference from the ordinary least squares (OLS) regression models of species richness against phylogenetic predictors for Theaceae and Theeae.

Table S4 Multimodel inference from the ordinary least squares (OLS) regression models of phylogenetic structure (NRI) against environmental predictors for Theaceae and Theeae.

Figure S1 Normal Q-Q plots for the ordinary least squares (OLS) regression models. For OLS models at the family level (i.e. Theaceae),

a: richness~MINT+MAP+MAP^2^

b: richness~MINT+MINT^2^+pH+pH^2^

c: richness~NRI

d: NRI~MINT+MAP

e: NRI~MINT+pH.

For OLS models at the tribe level (i.e. Theeae),

f: richness~MINT+MAP+MAP^2^

g: richness~MINT+MINT^2^+pH+pH^2^

h: richness~NRI+NRI^2^

i: NRI~MINT+MINT^2^+MAP^2^

j: NRI~MINT+MINT^2^+pH+pH^2^.

Figure S2 Relations of species richness with minimum temperature of the coldest month (MINT) and mean annual precipitation (MAP) in the ordinary least squares (OLS) regression models. SRF indicates species richness at family level (i.e. Theaceae), SRT indicates species richness at tribe level (i.e. Theeae). Species richness responses to each predictor in multiple models were showed, holding all other predictors constant. The regression line is given in blue, 95% confidence intervals is given in grey. Multimodel inference results are given in Table S2.

Figure S3 Relations of net relatedness index (NRI) with minimum temperature of the coldest month (MINT) and mean annual precipitation (MAP) in the ordinary least squares (OLS) regression models. NRIF indicates NRI at family level (i.e. Theaceae), NRIT indicates NRI at tribe level (i.e. Theeae). NRI responses to each predictor in multiple models were showed, holding all other predictors constant. The regression line is given in blue, 95% confidence intervals is given in grey. The scatter plot, with no relationship is also shown. Multimodel inference results are given in Table S4.
